# Perceptual Experiences of Autistic People With an Intellectual Disability and People With Williams Syndrome: A Reflexive Thematic Analysis

**DOI:** 10.1111/jar.13326

**Published:** 2024-11-29

**Authors:** Freya Elise, Brian Irvine, Jana Brinkert, Charlie Hamilton, Emily K. Farran, Elizabeth Milne, Gaia Scerif, Anna Remington

**Affiliations:** ^1^ Centre for Research in Autism and Education (CRAE), Department of Psychology and Human Development IOE, UCL's Faculty of Education and Society London UK; ^2^ Department of Psychological Sciences School of Psychology, University of Surrey Guildford UK; ^3^ Department of Psychology University of Sheffield Sheffield UK; ^4^ Department of Experimental Psychology University of Oxford Oxford UK

**Keywords:** attention, autism, distractibility, focus, intellectual disability, perception, perceptual capacity, Williams Syndrome

## Abstract

**Background:**

Autistic people without intellectual disabilities have increased perceptual capacity: they can process more information at any given time compared to non‐autistic people. We examined whether increased perceptual capacity is evident across the autistic spectrum (i.e. for autistic people with intellectual disabilities) and whether it is specific to autism, or also experienced by people with Williams Syndrome (WS).

**Methods:**

Five autistic adults with intellectual disabilities and five adults with WS took part in accessible, qualitative interviews. Responses were analysed using thematic analysis.

**Results:**

Both groups expressed enjoyment of focussed attention, with autistic participants preferring multiple simultaneous inputs. Responses suggested increased perceptual capacity for autistic participants only. The sensory environment was reported to be anxiety‐inducing for both groups.

**Conclusions:**

This study gives preliminary evidence that increased perceptual capacity may be universal across the autistic spectrum, and specific to autism. Understanding differences in capacity offers more targeted suggestions to support sensory challenges.

## 1 Introduction

Autism is a neurodevelopmental condition diagnosed based on differences in social communication and rigid and repetitive behaviour and routines (American Psychiatric Association 2013). Within the demands of a neurotypical world, these differences can prove challenging, and lead to difficulties in being understood by the non‐autistic majority (Happé and Frith 2020). In addition to these social and non‐social differences, studies indicate that autistic people have different perceptual experiences compared to non‐autistic people. These differences span a range of hyper‐ and hypo‐sensitivities, and are thought to be experienced by more than 90% of autistic people at some point in their lives (Crane, Goddard, and Pring 2009; Leekam et al. 2007). For example, we have shown that autistic people have greater perceptual capacity: they are able to process more visual and auditory information at any one time (e.g. redacted for review). This finding has been seen in lab‐based tasks of attention (e.g. O'Riordan et al. 2001; Remington and Fairnie 2017; Remington, Swettenham, and Lavie 2012) and is also beginning to emerge in autistic people's first‐hand qualitative accounts of perceptual experiences (Irvine et al. 2024). Indeed, increased capacity can offer an explanation for both positive and negative aspects of the autistic experience. For example, when engaging with a task with high perceptual load (i.e. a task which involves a great deal of potentially task‐relevant information), having increased perceptual capacity can allow more effective processing of the necessary information and, consequently, superior task performance (Remington, Swettenham, and Lavie 2012). In contrast, when engaging with a low perceptual load task (where task demands do not fill one's perceptual capacity), having extra capacity can have a negative impact as it may result in the automatic processing of task‐irrelevant information, resulting in increased vulnerability to distraction (Remington et al. 2009).

All these studies, however, have exclusively involved autistic people without an intellectual disability.^1^ What is not yet known is whether increased perceptual capacity is shared by autistic people with intellectual disabilities. Whilst not part of the core diagnostic criteria, the extent of co‐occurring intellectual disability in autistic people has previously been suggested to be as high as 50%–70% (Fombonne 2009), although more recent estimates are closer to 30% (Christensen et al. 2016). Within the United Kingdom, these rates vary across regions, age groups and socio‐economic groups (O'Nions et al. 2023). For example, 41% of older autistic adults (aged 50–59 years) in England also have an intellectual disability (likely due to historical trends where autistic people without an intellectual disability were overlooked with respect to diagnoses), in contrast to 4%–11% of those below the age of 19 years (O'Nions et al. 2023). Despite what may be a sizable percentage of autistic adults also having an intellectual disability, the majority of autism research has not included these individuals. A meta‐analysis revealed that 82% of studies showed a selection bias against participants with an intellectual disability despite applying their findings to the entire autistic spectrum (Russell et al. 2019). Further, even within those studies that include autistic people with an intellectual disability as participants, their voices are rarely represented (Wilson et al. 2020). An exclusion of this type risks neglecting a significant subsection of the population, undermining their right to understanding, appropriate services, and evidence‐based support. To truly assess the universality of increased perceptual capacity in autism, it is necessary to extend research in this area to consider individuals across the entire autistic spectrum. This should include those with intellectual disabilities and their first‐hand experiences.

A second unanswered question regarding increased perceptual capacity is the extent to which this is specific to autistic people or is shared by people with other neurodivergent conditions. In a first step towards answering these two key questions regarding the universality and specificity of increased perceptual capacity, the present study examines the perceptual experiences of a group of autistic people with intellectual disabilities and a group of people with Williams Syndrome (WS).

WS is a rare genetic condition that affects between 1 in 7500 (Strømme, Bjømstad, and Ramstad 2002) and 1 in 20,000 (Morris et al. 1988) people. It is due to a deletion on chromosome 7, which leads to distinctive facial characteristics, a wide range of learning difficulties and physical health problems (Donnai and Karmiloff‐Smith 2000). There are a number of similarities between the experiences of autistic people and those with WS. Like many autistic people, those with WS often experience sensory processing differences. For example, hyperacusis (increased sensitivity to sound) and phonophobia (distress in response to certain sounds) affects almost all people with WS (see Metcalfe 2012). Similarly, there is a higher likelihood of inattention and distractibility compared with the general population (Rhodes et al. 2011). Though there are many commonalities between the two conditions, people with WS are often characterised as talkative and friendly, in ways that can sometimes cause difficulties (Royston et al. 2021) whereas autistic people often experience challenges initiating social communication (Baird and Norbury 2016). As such, it is important to establish whether people with WS share an increased perceptual capacity, or if their sensory experiences are rooted in an alternative aetiology. Comparing these two specific groups will reveal whether increased perceptual capacity is autism‐specific, or experienced by all those who encounter sensory sensitivities.

Indeed, understanding individual differences in perceptual capacity (both within and across neurotypes) is crucial because of the practical implications that follow. Reframing differences in attentional experiences in terms of increased capacity rather than a failure of attentional control (Bayliss and Kritikos 2011) offers specific suggestions regarding the best way to support attentional challenges. For example, those with increased perceptual capacity may benefit from tasks that involve a *greater* amount of information (rather than a simplified version). Engaging more of one's perceptual capacity with task‐relevant or neutral information can be helpful because it reduces the risk of extra capacity being used to process distracting elements (Remington et al. 2019). Though we view the construct of attention in multi‐faceted ways, and subscribe to multiple, non‐mutually exclusive models (e.g. Amso and Scerif 2015) the present work is centred on the framework offered by Load Theory of Attention and Cognitive Control (Lavie 2005). This is because it has previously helped to elucidate autistic attentional experiences (e.g. Remington, Swettenham, and Lavie 2012). Load Theory posits that task‐irrelevant sensory processing is dependent on the amount of potentially‐task relevant information involved in a given task (‘perceptual load’), and the extent to which it fills one's perceptual capacity. As noted above, individual differences in this perceptual capacity can have both positive and negative practical implications.

In the present study, we seek to present first‐hand perceptual experiences of autistic people with intellectual disabilities and those with WS. Within these experiences, we aim to identify aspects that might suggest greater perceptual capacity for either of these participant groups. To do this, we created an accessible and adaptive interview protocol to meaningfully include participants with intellectual disabilities and answer the following research questions:

1. What are the similarities and differences between how autistic adults with intellectual disabilities and adults with WS experience processing of information, with a specific emphasis on experiences of focus and distraction?
2. To what extent can the accounts of information processing, focus and distraction given by autistic people with intellectual disabilities and those with WS be interpreted as evidence of increased perceptual capacity?

## 2 Materials and Methods

### 2.1 Community Involvement

The present study was conducted by a group of autistic and non‐autistic researchers. However, whilst members of the team are multiply‐neurodivergent, none have an intellectual disability or WS. As such, we sought additional input from those with intellectual disability and the WS community. Before taking part in the study, three adults with WS and three autistic adults with intellectual disabilities (recruited via UK‐based charities) acted as paid advisors to the project during the design phase of the study. Advisors provided this consultation in short online sessions, via Zoom, with support from a Trusted Adult (also paid for their time) which ranged from technical support, to being on the call but not contributing. Each advisor took part in between one and four sessions, with most doing two or three sessions. The advisors gave feedback on the protocol for study participation. This included the design of the Research Passport (see below), how participants could be supported by a Trusted Adult, how they chose their Trusted Adult, the interview rubric, including personalising interview questions and the recruitment materials (i.e. participant information sheet, recruitment videos and consent form). To elicit recruitment feedback specifically, advisors discussed the following with the team: what it means when deciding to take part in research, information they want in order to make that decision and the formats for that information.

### 2.2 Participants

Participants in the present study were 10 UK‐based adults (over 18 years of age) with an intellectual disability. Five participants were autistic, and five participants had WS. All had received clinical diagnoses from an independent clinician. Two autistic participants also had a diagnosis of ADHD, and participants from both groups had various other conditions (e.g. anxiety/depression). To protect anonymity in light of the small participant numbers, full details of additional health conditions are not presented. Each person took part in the study with a Trusted Adult of their choice who was a parent, carer, sibling or workplace mentor. Participants were recruited via UK‐based charities (Williams Syndrome Foundation, Mencap, Autistica), social media and the researchers' own networks. When potential participants – or someone who supported them –expressed an interest via email, we sent them an explanatory video, an accessible information sheet and an adapted consent form. All participants were able to provide informed consent to take part in the research via these materials.

The autistic group was 60% male (assumed gender), with a mean age of 35.0 years (range = 19–58 years; SD = 14.8). All autistic participants had support in their daily lives, with three participants living in their family homes, one participant in specialist‐supported housing for people with intellectual disabilities and one participant supported in their own flat. One autistic participant attended a college for people with intellectual disabilities and three were in part‐time supported employment.

Participants with WS were 40% male (assumed gender), with a mean age of 30.8 years (range = 28–36 years; SD = 3.19), and also received a range of care and support. Three participants lived in their family home, one was in specialist‐supported housing and one lived independently. Four participants with WS were in voluntary work multiple days per week. All participants with WS were regularly involved in a range of activities, predominantly via groups specifically for adults with intellectual disabilities.

Participants over 25 (*n* = 8) had support from adult social care to access volunteer work and their community. Participants under 25 (*n* = 2) were accessing post‐19 specialist college via Education Health Care Plans.

Standardised tests (see Section 2.3) confirmed that participants performed at a level consistent with an intellectual disability (i.e. IQ below 70), although one participant from each group declined to participate in this part of the study. One found it too stressful, and one declined due to having done them many times previously. Autistic participants scored between the 2nd and 16th percentile (*M* = 8.3) on the British Picture Vocabulary Scale third edition (BPVS‐3, Dunn and Dunn 2009) and from the 1st to 63rd percentile (*M* = 32.3) on the Ravens Coloured Progressive Matrices (RCPM, Raven 1962). Participants with WS scored between the 2nd and 96th percentile (*M* = 37.5) for the BPVS‐3^2^ and from below the 1st percentile to the 5th percentile (*M* = 1.6) for RCPM.

### 2.3 Measures

#### 2.3.1 Interview Schedule

A bespoke interview schedule was developed for the present study. Questions were based on a previous qualitative approach to exploring the attentional experiences of neurodivergent adults without intellectual disability (Irvine et al. 2024). The original schedule included questions about focus and distraction (e.g. ‘Do you like doing one thing at a time or lots of things at once?’). These were asked using concrete examples to ground each point (e.g. What are you doing when you watch x? Where do you watch it? Do you wear headphones? etc.). In this study, this scaffolding was personalised in advance based on information provided in each participant's Research Passport (see Section 2.3.2). For an example interview schedule, see Data S1. Interviews were conducted via Zoom or in person in the participants' own home (to minimise physical access barriers), and took approximately 30 min (range: 20–50 min) to complete. Support from a Trusted Adult during these interviews ranged from technical support, to being present but not contributing, to being actively involved in the conversation. All types of input were seen for both participant groups. Conversations were audio recorded (and subsequently transcribed) with additional notes made by hand/typed for clarity. Non‐verbal responses such as thumbs up or shaking of the head were clarified via researcher speech and noted as a yes or no answer. Even where participants gave only few verbal responses, they were able to indicate yes or no responses to which their Trusted Adult provided examples or further information that the participant agreed with or disagreed with for example nodding or signing ‘stop’ to disagree with the Trusted Adult's view, example or interpretation. The content, rather than mode of communication, was analysed. In this way we ensured that the input of less verbal individuals was given due weight in the analysis. Likewise, great care was taken to confirm that participants were discriminating in their responses, rather than expressing blanket agreement which might suggest a lack of volition.

#### 2.3.2 Research Passport

The bespoke Research Passport used in this study was developed based on the Camden Traffic Light Booklet – Hospital Passport, which is used to gain information about an individual's likes, dislikes, preferences for communication and daily life (Camden Traffic Light Booklet n.d.). It is similar in aims to the Research Passport for Autistic Adults without intellectual disability developed by Ashworth et al. (2021). The document used in the present study included simple language and pictures and could be completed on a computer, tablet or paper. The passport enabled the researchers to understand the individual before they began working together to make the research process as positive as possible. For example, learning about an autistic participant's preferred daily routine, which – if interrupted – may cause distress. The questions in the research passport were tailored to the study requirements of the present interview‐based study and therefore focussed on communication and planning rather than – for example – physical access needs. The interviewer also completed and shared their own Research Passport so that participants also got to know the researcher. As this Research Passport had not been used before, we sought feedback on its suitability from our study advisors with intellectual disabilities (both autistic and those with WS) before using it in the wider study. The advisors endorsed all aspects of the Research Passport, and therefore no changes were needed (Elise 2022).

The content of the participants' Research Passports allowed us to adapt the initial interview rubric to each individual, making questions specific to their everyday activities and experiences. Additionally, the Research Passport gave us initial information about how the participant communicated with people on a day‐to‐day basis (e.g. how they would indicate to researchers that they wanted to stop/they needed a break).

#### 2.3.3 Standardised Measures

Standardised measures of ability were included in order to characterise our participant group, allowing the relevance and generalisability of our findings to other population groups to be assessed. Due to the frequent exclusion of autistic people with intellectual disabilities from research, we also wanted to use standardised measures to evidence the inclusion of autistic participants with intellectual disabilities. A diagnosis of intellectual disability is typically made in childhood and is defined by an IQ score below 70, and difficulties in adaptive functioning (MacKay 2009). We selected two measures for their ability to capture two different domains (receptive language and non‐verbal reasoning) without participant burden of a full IQ battery. We expected that participants with WS would perform relatively well in the language‐based domain, and autistic participants would be more likely to perform relatively well in a non‐verbal‐based domain.

The RCPM (Raven and Raven 2003) is a standardised task widely used to assess non‐verbal reasoning. RCPM includes 36 trials divided into three subtests of 12 trials each. In each trial, the participant is presented with a coloured pattern where one part is missing, and the participant is asked to select the missing part from six options. In each sub‐test, the trials are ordered by increasing difficulty. There is no set time limit to complete trials. RCPM has been shown to be a valid tool with participants with WS (Van Herwegen, Farran, and Annaz 2011) and is particularly accessible for many autistic participants (Dawson et al. 2007; Soulières et al. 2009). The BPVS‐3 is a multiple‐choice assessment of standard English receptive vocabulary (Dunn and Dunn 2009). Devised for use with individuals aged 3–16 years, reliability is reported as 0.91 and validity with the Wechsler Intelligence Scale for Children as *r* = 0.76. Administration of the BPVS involves the researcher saying a word and the participant responding by pointing to a picture (from four options) that best illustrates the meaning of the word. The BPVS has been used extensively in research with autistic people, those with WS and people with intellectual disabilities (e.g. Annaz et al. 2011; Purser et al. 2015; Rhodes et al. 2011; Startin et al. 2019).

The standardised measures were presented online via Zoom (*n* = 6), or in person (*n* = 2), depending on preference. Two participants – one autistic and one with WS – chose not to complete the standardised measures. Where the administration was remote, participants selected their answer by pointing, and they or their Trusted Adult then conveyed the numeric answer. We did not expect any difference in RCPM scores due to the variety of administration settings; however, we were mindful that Ashworth et al. (2021) found a slight elevation in BPVS‐3 scores for those tested virtually compared to those tested in person. This was not a concern for the present study, as we only used the standardised scores to confirm intellectual disability rather than a key outcome measure.

### 2.4 Protocol

Ethical approval was granted by the IOE, UCL's Faculty of Education and Society's Ethics Committee, and all participants provided informed consent before beginning the study.

#### 2.4.1 Advance Familiarisation

Once participants had consented, we sent them a Research Passport along with completed Research Passports for the researcher(s) they would meet. Depending on preference, these were provided as printable email attachments or fillable PDFs or sent in paper format with a self‐addressed envelope via post. Participants and/or their Trusted Adult then communicated with the research team, as needed, to return the Research Passport and agree on times/dates for the next steps. Every participant's Trusted Adult was involved in the scheduling of the meetings, was copied into all emails and was present at the time of the Zoom call (virtually, on their own device or in the same room or an adjoining room to the participant). Researchers offered a range of options to familiarise the participants with Zoom, the researcher themself and the standardised measures. Some participants requested introductory videos of the researcher they would meet, brief Zoom calls to say hello and communicate about agreed interests, visual information about the interview, an example of the BPVS and an example of the RCPM.

#### 2.4.2 Study Measures

Those who took part remotely (*n* = 8) completed the interview in one session and then performed the standardised measures on a different day. Some participants had additional thoughts that they wanted to contribute after the interview had finished, and shared these via email, told us when we met online for the standardised measures session, or scheduled an additional Zoom. Those who were visited in their own home (*n* = 2) did all aspects of the study (interview and standardised measures) in 1 day. Participants were given full flexibility about how this could be organised across the day.

Prior to conducting the standardised measures, the researchers communicated with the participant and/or their Trusted Adult to ascertain if the participant would find the researcher saying ‘great’/‘well done’ or similar statements difficult, or if the absence of these statements or them only being said periodically would cause distress. Many, but not all, participants expressed that they liked praise. Where necessary, participants could move around throughout the testing sessions to facilitate comfortable engagement. This was achieved via the use of tablet devices, multiple devices or a wide‐angle camera set up.

#### 2.4.3 Post‐Study Communication

Following the testing sessions, participants were paid for their time and sent a thank you card or email and/or had a goodbye Zoom call. We also continue to update participants regarding the study outputs.

### 2.5 Analysis

Scores on the standardised IQ measures were analysed descriptively. We used Reflexive Thematic Analysis to analyse the qualitative interview responses (Braun and Clarke 2022a). We employed a hybrid inductive/deductive approach to the thematic analysis (Byrne 2022). Our analysis was primarily inductive, to prioritise the lived experiences of those we spoke to, and ensure that the experiences of those with learning disabilities were truly reflected – rather than being in any way curtailed by our norms, given that none of the research team has an intellectual disability (Braun and Clarke 2022b). However, we also drew on our previous work on attentional differences across neurotypes (Irvine et al. 2024) as a deductive framework to identify aspects of focus, distraction and perceptual capacity. Our analysis was informed by our positionalities as a neurodiverse research team (a collection of researchers who are autistic, non‐autistic and have other areas of neurodivergence), and by our endorsement of a ‘social model plus’: a social model of disability that acknowledges both the disabling nature of neurotypical societal norms, and the embodied disablement of being neurodivergent (Shakespeare 2014).

The coding was led by FE, in collaboration with AR, using Braun and Clarke's six steps for reflexive thematic analysis (Braun and Clarke 2022a). Once all interviews were complete, FE familiarised themselves with the data and shared first insights and introspections with respect to their own positionality with AR. Coding was done on the entire interview set together, rather than separately for each group. Discussions between FE and AR led to initial themes/categorisation and further refinement, then led to the presented themes and subthemes. All authors reviewed and approved the final set of themes.

## 3 Results

**FIGURE 1:**
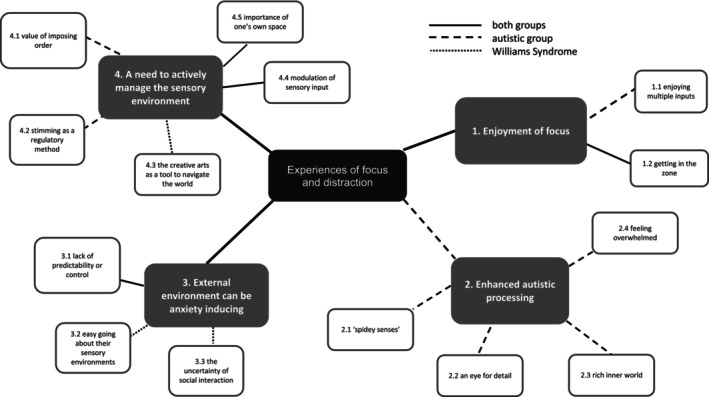
Diagram of themes and sub‐themes. Line style indicates when themes were unique to a particular diagnostic group or shared by both groups.

Using reflexive qualitative analysis, we identified four main themes and 14 sub‐themes from the data (see Figure 1 for the thematic map, and Table 1 for additional example quotes). Data from autistic participants and those with WS are considered together below, but each quote is identified by participant ID and group, in order to highlight group overlap or divergence and to show the spread of responses. We note, however, that any comparisons should be considered tentative due to the small sample sizes and potential selection bias that may result from the targeted sampling methodology. In cases where a theme was identified in both diagnostic groups, we have included one example quote in the text to illustrate each point, but have presented equivalent quotes from the other neurotype group in Table 1. We have replaced the original pronouns with gender‐neutral pronouns in all quotes, to further protect anonymity. Whilst the small participant numbers prevent the creation of a separate group of autistic people with ADHD (distinct from the autistic participants without ADHD), we comment on one subtheme where nuanced differences were noted for those with ADHD compared to the other participants. In all other themes and subthemes, similar sentiments were shared by autistic people with and without ADHD.

**TABLE 1:** Table of themes.

		Illustrative quotes	
	Sub‐themes	Autistic group	Williams Syndrome group
1. Enjoyment of focus	1.1 Enjoying multiple inputs	Does paper word puzzles whilst watching TV quiz shows [Aut‐C]	N/A
	1.2 Getting in the zone	‘Yes, I'd do it all the time. All day’ [Aut‐A]	Participant: ‘Couple of hours. Yeh, couple of hours just chill.’ Trusted Adult: ‘She's really very good at concentrating if she is enjoying it she could be there for about an hour’ [WS‐B]
2. Enhanced autistic processing	2.1 Spidey senses	‘Sees dangerous things better than others’ [Trusted Adult of Aut‐B]	N/A
	2.2 An eye for detail	‘Notices volume change for adverts, hears cars going past when watching TV’ [Trusted adult of Aut‐B]	N/A
	2.3 Rich inner world	[talking about reading] ‘I can see the castle and the forests and the moon. I'm looking down on the forest from somewhere high up like a castle, I see the trees just the colour dark green with the leaves. If I'm in the forest I can see the forest I can see the trees individually’ [Aut‐E]	N/A
	2.4 Feeling overwhelmed	‘Really irritating to have two people trying to talk to you. Something I get confused’ [Aut‐C]	N/A
3. The external environment can be anxiety‐inducing	3.1 Lack of predictability or control	‘By myself I get more anxious and worried. Anxious about something happening’ [Aut‐E]	‘It's sudden noise…it is the unpredictable nature of it’ [WS‐B]
	3.2 Easy going about their sensory environment	N/A	Part of life; ‘just get on with it’ (other people's noise) [WS‐C]
	3.3 The uncertainty of social interaction	N/A	‘Are they going to go bitching behind my back if I say something wrong, like make quick assumptions’ [WS‐A]
4. A need to actively manage the sensory environment	4.1 Value of imposing order	‘We need to know things in advance… I like to call in advance and ask “when do you get quiet times?” “Is there anywhere we can sit that is away from everywhere?”’ [Trusted adult of Aut‐D]	N/A
	4.2 Stimming as a regulatory method	‘Calming things like my [fidget] toys’ [Aut‐A]	N/A
	4.3 The creative arts as a tool to navigate the world	N/A	‘We sing out own songs. We create our own songs’ [WS‐A]
	4.4 Modulation of sensory input	‘On and off noise cancellation’ [Aut‐A]	‘Might get ear muffs to help’ [WS‐E]
	4.5 Importance of one's own space	‘spacious is good…have my own personal space’ [Aut‐E]	‘I knew I could live at my flat because I could see where my things would go’ [WS‐E]

### 3.1 Enjoyment of Focus

Participants in both groups shared examples of where they enjoyed paying attention to their passions, hobbies and volunteer jobs: ‘Yes, I'd do it all the time. All day’ (Aut‐A). The situations that afforded this focus appeared to differ slightly between the groups. For example, many autistic participants reported *enjoying multiple inputs* simultaneously: ‘playing with the DS [handheld games console] whilst watching something on AllFour [ondemand TV streaming service]’(Aut‐A). For the participants with ADHD, this went beyond enjoyment to a need: ‘I think [they are] always doing multiple things….[they] have to have something going on in the background…I think, that is the only way I can get [them] to concentrate’ [Trusted Adult of Aut‐D]. Whilst some participants with WS also mentioned having background noise whilst paying attention to a task, this was usually happenstance rather than by design. For example, when asked whether they did anything else at the same time as watching videos, WS‐B answered ‘I just enjoy it to be honest, which is lovely’.

In addition to the multi‐tasking, a number of participants from both groups shared their experiences of *getting in the zone*: paying attention to one topic whilst excluding all others: ‘Get into the zone. Stop thinking about disability, negatives, focus on the now’ [WS‐C]. Though the theme was present for autistic participants and those with WS, there was a nuanced element to the experience that somewhat separated the groups. For autistic participants, this involved a level of hyperfocus that meant less sense of time passing or other stimuli: ‘not really a limit…I watch it all the time…time just goes’ [Aut‐C]. Conversely, those with WS maintained more of a link with the external environment and retained an awareness of bodily functions: ‘I get hungry, look at the clock and then go make lunch’ [WS‐E]. This meant they were more susceptible to distraction even whilst in a state of focus. For example, when asked if it was easy to get their attention when in a state of focus, WS‐C answered, ‘it is’. Through all the examples given by our participants, there was a distinction between the ability to focus on tasks and topics that were of interest, and of their own volition, compared to experiences of paying attention to tasks/topics when asked by others. Participants with WS were generally happy to do household chores and jobs they may not have a preference for, but most needed support to stay on task: ‘I need quite a lot of help to do tasks…I think I get distracted because my mind kind of wanders off, my brain's kind of like “I can't focus anymore”’ [WS‐A]. Others were only able to engage with tasks they enjoyed: ‘I think [they] probably wouldn't do it. [They]’d avoid’ [Trusted Adult of WS‐B]. Autistic participants needed more specific supports to engage in tasks: ‘[They] have to have something going on in the background…the only way I can get [them] to concentrate…and it might just be anything as simple as helping me take a few things upstairs…and [they] can…as long as I give [them] instructions…as long as [they]’d got this [something playing on headphones]’[Trusted adult of Aut‐D].

### 3.2 Enhanced Autistic Processing

In a theme unique to the autistic participants, were several reports of enhanced perceptual processing. These fell broadly into four types of experience. The first considered times when autistic participants felt heightened ‘*spidey senses*’ that allowed them to be hyper‐vigilant ‘…like a sixth sense…I notice everything around me even when I'm lifting weights’ [Aut‐E].

Second, this enhanced processing meant that autistic participants felt they had *an eye for detail* and noticed things that others might miss: ‘I listen to them, but also listen to all the things around me. Like I can hear what everyone else is talking about and doing’ [Aut‐A].

This extra processing in the external environment was mirrored by a *rich inner world* where participants shared experiences of vivid imagination: ‘when I'm on an exercise bike I just close my eyes and pretend I'm somewhere else, like in the mountains somewhere warm and nice. I can see it in my head. Places in Spain and France’ [Aut‐E]. The fourth type of experience was less positive; participants explained how this increased processing could result in *feeling overwhelmed*: ‘too much environmental input impacts [them] and then [they] just drop to the ground and isn't thinking about where that is’ [Trusted adult of Aut‐D].

### 3.3 The External Environment Can Be Anxiety‐Inducing

Participants in both groups shared examples of ways in which external stimuli could lead to anxiety. There were, however, nuanced differences in these experiences depending on the diagnostic group. For example, whilst participants in both groups described how sensory‐related anxiety was linked to a *lack of predictability or control* over the situation, the group experiences were distinct. Autistic participants expressed a more overarching need for certainty within their daily lives: ‘I've heard things of routine, and it's set. And it breaks with changes that… that gets my head confused and annoyed. [Shows clenching body and arms tight and shaking] “Stressful”’ [Aut‐A]. Conversely, the majority of participants with WS had anxiety about the very specific situation of unpredictable, sudden onset, loud noises: ‘Surprise noises like tractors at work at the farm’ [WS‐C]. For some this led to fear and avoidance of activities that may involve these unpredictable noises, such as a dog (who may bark), motorbikes and spending time with family: if ‘there is the two of us [mum and dad] together [WS‐B] will disappear because [they] are worried that we might laugh’ [Trusted adult of WS‐B]. For others it caused anxiety related to hobbies or tasks that they enjoyed: ‘If I'm at a gig then there is pyrotechnics, I think I'm going to go home and leave but I think it is my anxiety… Pyrotechnics are surprising’ [WS‐C]. The detrimental outcome of being exposed to these sudden, loud noises was clear: ‘Anxiety that leads to panic attack, feels like a tidal wave in my brain and then sweat, scratch my head. I know I'll be ok afterwards but I still have to focus on getting through it’ [WS‐C].

Despite these specific examples of anxiety, participants with WS were in many ways very *easy‐going about their sensory environments*. For example, feeling relaxed about people putting on the radio in the background, or selecting a channel they had not chosen: ‘I still like it. I don't get annoyed’ [WS‐B]; ‘I don't mind [if housemate puts radio on]’ [WS‐D].

One additional source of anxiety for participants with WS centred on *the uncertainty of social interaction*, and awareness of their own differences within society: ‘What do people think of me? What do they actually think of me, and do they genuinely care?’ [WS‐A]. These worries appeared not to be expressed by our autistic participants.

### 3.4 A Need to Actively Manage the Sensory Environment

In keeping with the various negative experiences, our participants shared how navigating the world around them involved a need to actively manage the sensory environment, rather than passively receive sensory input. This management spanned a number of domains and approaches. Autistic participants, but not those with WS, shared examples of the *value of imposing order*: ‘Set timers and use a visual timetable…need to know things in advance’ [Aut‐D‐Trusted Adult] and *stimming as a regulatory method*: ‘calming things like my [fidget] toys’ [Aut‐A].

Many participants with WS used *the creative arts as a tool to navigate the world*, explaining how a love of poetry, music and art helped them stay focused and avoid negative rumination: ‘Sing stuff to get jobs done. I can come up with a song for any occasion’ [WS‐C].

For both groups of participants, the *modulation of sensory input* was crucial. This spanned both the visual (‘good lighting that you can adjust’ [Aut‐E]) and auditory domains: ‘took hearing aids out and used ear defenders’ [WS‐C].

Likewise, both autistic participants and those with WS explained the *importance of one's own space*. However, for autistic participants, this was mainly about the ability to ensure tranquillity; ‘…the thing that I need is, some space, because I don't want to get overwhelmed’ [Aut‐A]. Whereas for participants with WS, the value of the space was partially about comfort (‘I know where I am in the room’ [WS‐C]), but also connected to a sense of ownership and respect: ‘we feel respected…having a chair…means so much to me because when I was younger… they wouldn't let me sit down…it makes me very very proud to sit on my favourite chair’ [WS‐C].

## 4 Discussion

In the present study, we explored the daily perceptual experiences of a group of people with intellectual disabilities, half of whom were autistic and half who had WS. Including the voices of these participants is crucial in a research field which almost exclusively focuses on people who are cognitively and verbally able, and therefore risks neglecting the most vulnerable members of the neurodivergent community (Correia et al. 2017; Stedman et al. 2019; Tager‐Flusberg and Kasari 2013). Our participants discussed the situations in which they enjoyed paying attention to things, the aspects of the sensory environment that were more challenging, and strategies that they used to manage those challenges. Autistic participants shared examples of times when they felt they were processing more information than others around them.

Though autistic participants and those with WS shared an enjoyment of focus, the qualitative nature of the experience varied slightly by group. Autistic people liked processing a number of task‐related inputs simultaneously, whereas participants with WS did not require or prefer multiple streams. In addition, in situations of intense focus, those with WS reported a retained awareness of bodily sensations, whilst autistic participants spoke about a more all‐consuming experience. This echoes previous research on the phenomenological experiences of focus for autistic people that highlighted the positive yet all‐encompassing nature of task immersion (Rapaport et al. 2023). This sense of ‘flow’, where autistic people reported focussing deeply on task‐related aspects to the exclusion of all other stimuli, also chimes with recent work on Monotropism (Murray, Lesser, and Lawson 2005). Monotropism is an autistic‐led theory of autism that suggests the defining characteristic of autism is a single‐channel, or ‘monotropic’ attentional style. It is not clear, however, how this fits with reports from our autistic participants regarding a preference for concurrently processing multiple inputs.

The distinction between the autistic and WS experience in the present study also furthers our understanding of perception in autism versus other neurodevelopmental conditions. Our own work on the first‐hand perceptual experiences of autistic people and people with ADHD, in contrast to the comparison here, revealed a similar pattern of deep task immersion for both groups (Irvine et al. 2024). This is an interesting contrast to previous work which – based on assessment of characteristics – suggests more of an overlap between WS and ADHD profiles of attention (Rhodes et al. 2011).

Of particular relevance to the a priori research questions of the present study, the accounts of our participants cast light on the question of whether increased perceptual capacity is experienced by autistic people with intellectual disabilities, and also shared by those with other conditions. Autistic participants spoke about various aspects of enhanced processing: hyper‐vigilance, an eye for detail and a rich inner world – all of which can sometimes lead to a sense of overwhelm. This, together with a preference for multiple sensory inputs, suggests that perceptual capacity might be increased for autistic people with intellectual disabilities but not those with WS. We note, however, that we cannot rule out the possibility that those with WS *do* experience increased perceptual capacity but did not verbalise their experience regarding this in the present study. As such, whilst further investigation is needed to directly measure the extent of perceptual capacity in these groups, our findings offer a preliminary suggestion that the increased perceptual capacity observed for autistic people without intellectual disability (e.g. Remington and Fairnie 2017; Remington, Swettenham, and Lavie 2012) extends to those autistic people with intellectual disabilities. This reframing of attentional differences in terms of enhanced capacity has practical implications with respect to supporting any perceptual challenges encountered. For example, whilst of course, being mindful of avoiding sensory overarousal, *adding* task‐relevant information may help aid focus by filling capacity rather than leaving spare capacity, which would result in irrelevant stimuli processing and may lead to greater distraction or rumination. This challenges the conventional wisdom of minimising complexity for autistic people with intellectual disabilities (Ashburner, Ziviani, and Rodger 2008).

Whilst many aspects of attention and perception were positive, both groups also experienced anxiety related to the sensory environment (Glod, Riby, and Rodgers 2019). For those with WS, this clearly mapped onto prior research linking the condition with hyperacusis (Metcalfe 2012): our participants spoke about distress caused by loud, sudden noises, such as fireworks and dogs barking. In some cases, however, those with WS were more relaxed about their physical environment, and instead concerned about social interaction; concerns which were not raised by the autistic participants. When considering how to manage the challenges, autistic participants spoke about strategies to impose order on an unpredictable environment (e.g. using timers) and how stimming was helpful to reduce anxiety. In contrast, participants with WS embraced the creative arts to help navigate the external environment. Both groups used techniques to modulate sensory input, including headphones, adjustable lighting and having their own space.

Our findings also demonstrated the crucial role of motivation. For both groups, there was a sense that one's ability to perform a task or engage with a situation was dependent on intrinsic motivation. Our participants' accounts are in line with longstanding research demonstrating the positive impact of motivation on performance for people with intellectual disabilities (see Sideridis and Scanlon 2006). Whilst this was not an aspect we had set out to examine, it has important implications for the perceived abilities of people with intellectual disabilities. For example, assessments most often comprise tasks that are not intrinsically motivating, and are undertaken based on external instruction rather than one's own volition. This may lead to an underestimation of abilities for this group. Research on this topic is in its infancy, due to widespread exclusion of those with intellectual disabilities from research (e.g. Tager‐Flusberg and Kasari 2013), however, similar findings have recently been reported for non‐speaking autistic people who reported being able to do more if they liked the task and had chosen to do it (Rose 2024).

The present study offers preliminary insight into the daily perceptual experiences of autistic people with intellectual disabilities and people with WS, however, the limitations of the findings should also be considered. The small sample size, whilst appropriate for qualitative work, limits the generalisability of the findings, and also precluded us from systematically assessing the influence that participants' other conditions (e.g. ADHD, mental health conditions) may have on their perceptual experiences. Relying on formal diagnoses, rather than screening for traits, may also have underestimated the level of co‐occurring conditions in both groups. Reiss, Levitan, and Szyszko (1982) described clinicians being unable to ‘see anything’ other than the intellectual disability when assessing patients with intellectual disability (i.e. diagnostic overshadowing). Indeed, guidance for assessing ADHD in adults with an intellectual disability was only published in a report by The Royal College of Psychiatrists (2021). In addition, we are drawing inferences about perceptual capacity without objectively measuring it. Future research should seek to create the necessary accessible tasks to systematically – and directly – assess perceptual capacity in those with intellectual disabilities. This would allow further investigation of findings from the present study that suggest subtle differences between the perceptual abilities and experiences of those with different neurodevelopmental conditions. However, the importance of considering qualitative experience alongside more objective lab‐based tasks should not be overlooked (Kenny, Remington, and Pellicano 2024). We also acknowledge that including Trusted Adults to support our participants may have influenced responses – either directly (if adults answered in place of participants) or if participants felt inhibited to respond in a certain way in their presence. To mitigate this risk, part of the recruitment process involved checking that all participants were able to give informed consent, and similarly to disagree/express their own opinion, even in the presence of their Trusted Adult. Further, the research team have prior experience working to elicit the views of people with intellectual disabilities and were therefore attuned to this issue and mindful that participants were expressing their own views. We note, however, that without the support of Trusted Adults, none of the participants in the present study would have felt comfortable taking part, and would have yet again been excluded from research.

## 5 Conclusions

Here we present the first account of the first‐hand perceptual experiences of a group of autistic people with intellectual disabilities and a group of people with WS. The findings point tentatively towards the universality of increased perpetual capacity across the autistic spectrum: autistic people with intellectual disabilities shared experiences that are suggestive of increased capacity, similar to previous findings of increased capacity for autistic people without intellectual disabilities. The nuanced differences in the perceptual experiences of autistic and non‐autistic people with intellectual disabilities seen in our study also suggest that this capacity increase may not be associated with all neurodevelopmental conditions. The negative aspects of sensory processing raised by these groups also underline the importance of considering the impact of the sensory environment, especially for those who might not always be able to advocate for themselves or articulate their own preferences.

## Author Contributions

**Freya Elise:** conceptualisation, methodology, formal analysis, investigation, writing – original draft, writing – review and editing. **Brian Irvine:** conceptualisation, methodology, investigation, writing – review and editing. **Jana Brinkert:** conceptualisation, writing – review and editing, funding acquisition, supervision. **Charlie Hamilton:** conceptualisation, writing – review and editing. **Emily K. Farran:** conceptualisation, writing – review and editing, supervision, funding acquisition. **Elizabeth Milne:** conceptualisation, writing – review and editing, supervision, funding acquisition. **Gaia Scerif:** conceptualisation, writing – review and editing, supervision, funding acquisition. **Anna Remington:** conceptualisation, formal analysis, writing – original draft, writing – review and editing, supervision, funding acquisition.

## Ethics Statement

All procedures were approved by the UCL IOE Research Ethics Committee and participants provided informed consent in advance of taking part, supported through co‐designed explanatory images and videos (Elise, Brinkert, and Remington 2022).

## Conflicts of Interest

The authors declare no conflicts of interest.

## Supporting information


**Data S1.** Supporting information.

## Data Availability

Quantitative data from this study are available from the authors on request. Due to ethical constraints, the qualitative data cannot be shared publicly.

## Endnotes

^1^
In the United Kingdom, ‘intellectual disability’ is often referred to as ‘learning disability’, the term generally preferred by the UK autistic community. However, in keeping with the journal's policy to use ‘learning disability’ only for certain contexts (where this refers to a specific learning disability/disorder—such as in reading or written expression), we use the term ‘autistic with an intellectual disability’. This refers to reduced intellectual ability (typically an IQ below 70) and difficulty with everyday activities (Mencap n.d.).

^2^
Whilst one participant scored extremely highly on the BPVS‐3 (at a level that is inconsistent with intellectual disabilities), this was accompanied by a very low RCPM score, aligned with the characteristic WS cognitive profile (Farran et al. 2024).

## References

[jar13326-bib-0001] American Psychiatric Association . 2013. Diagnostic and Statistical Manual of Mental Disorders: DSM‐5. 5th ed. Washington, DC: American Psychiatric Association.

[jar13326-bib-0002] Amso, D. , and G. Scerif . 2015. “The Attentive Brain: Insights From Developmental Cognitive Neuroscience.” Nature Reviews Neuroscience 16, no. 10: 606–619. 26383703 10.1038/nrn4025 PMC4885514

[jar13326-bib-0003] Annaz, D. , C. M. Hill , A. Ashworth , S. Holley , and A. Karmiloff‐Smith . 2011. “Characterisation of Sleep Problems in Children With Williams Syndrome.” Research in Developmental Disabilities 32, no. 1: 164–169. 10.1016/j.ridd.2010.09.008. 20940094

[jar13326-bib-0004] Ashburner, J. , J. Ziviani , and S. Rodger . 2008. “Sensory Processing and Classroom Emotional, Behavioral, and Educational Outcomes in Children With Autism Spectrum Disorder.” American Journal of Occupational Therapy 62, no. 5: 564–573. 10.5014/ajot.62.5.564 18826017

[jar13326-bib-0005] Ashworth, M. , O. Palikara , E. Burchell , H. Purser , D. Nikolla , and J. Van Herwegen . 2021. “Online and Face‐To‐Face Performance on Two Cognitive Tasks in Children With Williams Syndrome.” Frontiers in Psychology 11: 594465. 10.3389/fpsyg.2020.594465. 33613354 PMC7889503

[jar13326-bib-0006] Baird, G. , and C. F. Norbury . 2016. “Social (Pragmatic) Communication Disorders and Autism Spectrum Disorder.” Archives of Disease in Childhood 101, no. 8: 745–751. 26699538 10.1136/archdischild-2014-306944

[jar13326-bib-0007] Bayliss, A. P. , and A. Kritikos . 2011. “Brief Report: Perceptual Load and the Autism Spectrum in Typically Developed Individuals.” Journal of Autism and Developmental Disorders 41, no. 11: 1573–1578. 10.1007/s10803-010-1159-8. 21188489

[jar13326-bib-0009] Braun, V. , and V. Clarke . 2022a. Thematic Analysis: A Practical Guide. Thousand Oaks, CA: SAGE.

[jar13326-bib-0056] Braun, V. , and V. Clarke . 2022b. “Toward Good Practice in Thematic Analysis: Avoiding Common Problems and Be(com)ing a Knowing Researcher.” International Journal of Transgender Health 24, no. 1: 1–6. 10.1080/26895269.2022.2129597. 36713144 PMC9879167

[jar13326-bib-0010] Byrne, D. 2022. “A Worked Example of Braun and Clarke's Approach to Reflexive Thematic Analysis.” Quality & Quantity 56, no. 3: 1391–1412. 10.1007/s11135-021-01182-y.

[jar13326-bib-0011] Camden Traffic Light Booklet . n.d. University College London Hospitals NHS Foundation Trust. https://www.uclh.nhs.uk/patients‐and‐visitors/patient‐information‐pages/camden‐traffic‐light‐booklet.

[jar13326-bib-0012] Christensen, D. L. , J. Baio , K. V. N. Braun , et al. 2016. “Prevalence and Characteristics of Autism Spectrum Disorder Among Children Aged 8 Years—Autism and Developmental Disabilities Monitoring Network, 11 Sites, United States, 2012.” MMWR Surveillance Summaries 65, no. 3: 1–23. 10.15585/mmwr.ss6503a1. PMC7909709 27031587

[jar13326-bib-0013] Correia, R. A. , M. J. Seabra‐Santos , P. Campos Pinto , and I. Brown . 2017. “Giving Voice to Persons With Intellectual Disabilities About Family Quality of Life.” Journal of Policy and Practice in Intellectual Disabilities 14, no. 1: 59–67.

[jar13326-bib-0014] Crane, L. , L. Goddard , and L. Pring . 2009. “Sensory Processing in Adults With Autism Spectrum Disorders.” Autism 13, no. 3: 215–228. 10.1177/1362361309103794. 19369385

[jar13326-bib-0015] Dawson, M. , I. Soulières , M. Ann Gernsbacher , and L. Mottron . 2007. “The Level and Nature of Autistic Intelligence.” Psychological Science 18, no. 8: 657–662. 10.1111/j.1467-9280.2007.01954.x. 17680932 PMC4287210

[jar13326-bib-0016] Donnai, D. , and A. Karmiloff‐Smith . 2000. “Williams Syndrome: From Genotype Through to the Cognitive Phenotype.” American Journal of Medical Genetics 97, no. 2: 164–171. 10.1002/1096-8628(200022)97:2<164::aid-ajmg8>3.0.co;2-f. 11180224

[jar13326-bib-0017] Dunn, L. M. , and D. M. Dunn . 2009. The British Picture Vocabulary Scale. Windsor, UK: GL Assessment Limited, National Foundation for Educational Research.

[jar13326-bib-0018] Elise, F. , J. Brinkert , and A. Remington . 2022. Research Passport . 10.17605/OSF.IO/WQ43F.

[jar13326-bib-0019] Farran, E. K. , H. R. M. Purser , C. Jarrold , et al. 2024. “Cross‐Sectional and Longitudinal Assessment of Cognitive Development in Williams Syndrome.” Developmental Science 27, no. 1: e13421. 10.1111/desc.13421. 37287370

[jar13326-bib-0020] Fombonne, E. 2009. “Epidemiology of Pervasive Developmental Disorders.” Pediatric Research 65, no. 6: 591–598. 10.1203/PDR.0b013e31819e7203. 19218885

[jar13326-bib-0021] Glod, M. , D. M. Riby , and J. Rodgers . 2019. “Relationships Between Sensory Processing, Repetitive Behaviors, Anxiety, and Intolerance of Uncertainty in Autism Spectrum Disorder and Williams Syndrome.” Autism Research 12, no. 5: 759–765. 30919599 10.1002/aur.2096

[jar13326-bib-0057] Happé, F. , and U. Frith . 2020. “Annual Research Review: Looking Back to Look Forward – Changes in the Concept of Autism and Implications for Future Research.” Journal of Child Psychology and Psychiatry 61, no. 3: 218–232. 10.1111/jcpp.13176. 31994188

[jar13326-bib-0022] Irvine, B. , F. Elise , J. Brinkert , et al. 2024. “‘A Storm of Post‐It Notes’: Experiences of Perceptual Capacity in Autism and ADHD.” Neurodiversity 2: 27546330241229004. 10.1177/27546330241229004.

[jar13326-bib-0024] Kenny, L. , A. Remington , and E. Pellicano . 2024. “Everyday Executive Function Issues From the Perspectives of Autistic Adolescents and Their Parents: Theoretical and Empirical Implications.” Autism 28, no. 9: 2204–2217. 38240286 10.1177/13623613231224093 PMC11408970

[jar13326-bib-0025] Lavie, N. 2005. “Distracted and Confused?: Selective Attention Under Load.” Trends in Cognitive Sciences 9, no. 2: 75–82. 10.1016/j.tics.2004.12.004. 15668100

[jar13326-bib-0026] Leekam, S. R. , C. Nieto , S. J. Libby , L. Wing , and J. Gould . 2007. “Describing the Sensory Abnormalities of Children and Adults With Autism.” Journal of Autism and Developmental Disorders 37, no. 5: 894–910. 10.1007/s10803-006-0218-7. 17016677

[jar13326-bib-0027] MacKay, T. 2009. “Severe and Complex Learning Difficulties: Issues of Definition, Classification and Prevalence.” Educational and Child Psychology 26, no. 4: 9–18. 10.53841/bpsecp.2009.26.4.9.

[jar13326-bib-0028] Mencap . n.d. *What is a Learning Disability*? Mencap. Accessed August 31, 2022. https://www.mencap.org.uk/learning‐disability‐explained/what‐learning‐disability.

[jar13326-bib-0029] Metcalfe, K. 2012. “Clinical Profile: Diagnosis and Prognosis.” In Neurodevelopmental Disorders Across the Lifespan: A Neuroconstructivist Approach, 85–102. Oxford: Oxford Academic.

[jar13326-bib-0030] Morris, C. A. , S. A. Demsey , C. O. Leonard , C. Dilts , and B. L. Blackburn . 1988. “Natural History of Williams Syndrome: Physical Characteristics.” Journal of Pediatrics 113, no. 2: 318–326. 10.1016/S0022-3476(88)80272-5. 2456379

[jar13326-bib-0031] Murray, D. , M. Lesser , and W. Lawson . 2005. “Attention, Monotropism and the Diagnostic Criteria for Autism.” Autism 9, no. 2: 139–156. 10.1177/1362361305051398. 15857859

[jar13326-bib-0032] O'Nions, E. , I. Petersen , J. E. J. Buckman , et al. 2023. “Autism in England: Assessing Underdiagnosis in a Population‐Based Cohort Study of Prospectively Collected Primary Care Data.” Lancet Regional Health – Europe 29: 100626. 10.1016/j.lanepe.2023.100626. 37090088 PMC10114511

[jar13326-bib-0033] O'Riordan, M. A. , K. C. Plaisted , J. Driver , and S. Baron‐Cohen . 2001. “Superior Visual Search in Autism.” Journal of Experimental Psychology: Human Perception and Performance 27, no. 3: 719–730. 11424657 10.1037//0096-1523.27.3.719

[jar13326-bib-0034] Purser, H. R. M. , E. K. Farran , Y. Courbois , et al. 2015. “The Development of Route Learning in Down Syndrome, Williams Syndrome and Typical Development: Investigations With Virtual Environments.” Developmental Science 18, no. 4: 599–613. 10.1111/desc.12236. 25284087

[jar13326-bib-0035] Rapaport, H. , H. Clapham , J. Adams , W. Lawson , K. Porayska‐Pomsta , and E. Pellicano . 2023. “‘I Live in Extremes’: A Qualitative Investigation of Autistic Adults' Experiences of Inertial Rest and Motion.” Autism 28, no. 5: 1305–1315. 37776056 10.1177/13623613231198916 PMC11067417

[jar13326-bib-0036] Raven, J. C. 1962. Coloured Progressive Matrices, Sets A, A_B, B. Los Angeles, CA: Western Psychological Services.

[jar13326-bib-0023] Raven, J. , and J. Raven . 2003. “Raven Progressive Matrices.” In Handbook of Nonverbal Assessment, edited by R. S. McCallum , 223–237. Boston, MA: Springer US. 10.1007/978-1-4615-0153-4_11.

[jar13326-bib-0037] Reiss, S. , G. W. Levitan , and J. Szyszko . 1982. “Emotional Disturbance and Mental Retardation: Diagnostic Overshadowing.” American Journal of Mental Deficiency 86, no. 6: 567–574. 7102729

[jar13326-bib-0038] Remington, A. , and J. Fairnie . 2017. “A Sound Advantage: Increased Auditory Capacity in Autism.” Cognition 166: 459–465. 10.1016/j.cognition.2017.04.002. 28476357

[jar13326-bib-0039] Remington, A. , M. Hanley , S. O'Brien , D. M. Riby , and J. Swettenham . 2019. “Implications of Capacity in the Classroom: Simplifying Tasks for Autistic Children May Not Be the Answer.” Research in Developmental Disabilities 85: 197–204. 10.1016/j.ridd.2018.12.006. 30579260

[jar13326-bib-0040] Remington, A. , J. Swettenham , R. Campbell , and M. Coleman . 2009. “Selective Attention and Perceptual Load in Autism Spectrum Disorder.” Psychological Science 20, no. 11: 1388–1393. 10.1111/j.1467-9280.2009.02454.x. 19843262

[jar13326-bib-0041] Remington, A. M. , J. G. Swettenham , and N. Lavie . 2012. “Lightening the Load: Perceptual Load Impairs Visual Detection in Typical Adults but Not in Autism.” Journal of Abnormal Psychology 121, no. 2: 544–551. 10.1037/a0027670. 22428792 PMC3357114

[jar13326-bib-0042] Rhodes, S. M. , D. M. Riby , K. Matthews , and D. R. Coghill . 2011. “Attention‐Deficit/Hyperactivity Disorder and Williams Syndrome: Shared Behavioral and Neuropsychological Profiles.” Journal of Clinical and Experimental Neuropsychology 33, no. 1: 147–156. 20700845 10.1080/13803395.2010.495057

[jar13326-bib-0043] Rose, L. (Director). 2024, April 4. *They Need to Know That Non‐Speaking Doesn't Mean Non‐Thinking* [Video]. https://www.youtube.com/watch?v=I3yOn7mV0wk&ab_channel=CraeIOE.

[jar13326-bib-0044] Royal College of Psychiatrists . 2021. *Attention Deficit Hyperactivity Disorder (ADHD) in Adults With Intellectual Disability* (CR230). https://www.rcpsych.ac.uk/improving‐care/campaigning‐for‐better‐mental‐health‐policy/college‐reports/2021‐college‐reports/ADHD‐in‐adults‐with‐intellectual‐disability‐CR230.

[jar13326-bib-0045] Royston, R. , C. Oliver , P. Howlin , and J. Waite . 2021. “Anxiety Characteristics in Individuals With Williams Syndrome.” Journal of Applied Research in Intellectual Disabilities 34, no. 4: 1098–1107. 10.1111/jar.12864. 33561900

[jar13326-bib-0046] Russell, G. , W. Mandy , D. Elliott , R. White , T. Pittwood , and T. Ford . 2019. “Selection Bias on Intellectual Ability in Autism Research: A Cross‐Sectional Review and Meta‐Analysis.” Molecular Autism 10: 9. 10.1186/s13229-019-0260-x. 30867896 PMC6397505

[jar13326-bib-0047] Shakespeare, T. 2014. “Nasty, Brutish, and Short? On the Predicament of Disability and Embodiment.” In Disability and the Good Human Life, 93–112. Cambridge, UK: Cambridge University Press.

[jar13326-bib-0048] Sideridis, G. D. , and D. Scanlon . 2006. “Motivational Issues in Learning Disabilities.” Learning Disability Quarterly 29, no. 3: 131–135.

[jar13326-bib-0049] Soulières, I. , M. Dawson , F. Samson , et al. 2009. “Enhanced Visual Processing Contributes to Matrix Reasoning in Autism.” Human Brain Mapping 30, no. 12: 4082–4107. 10.1002/hbm.20831. 19530215 PMC2787806

[jar13326-bib-0050] Startin, C. M. , S. Hamburg , A. Strydom , and LonDownS Consortium . 2019. “Comparison of Receptive Verbal Abilities Assessed Using the KBIT‐2 and BPVS3 in Adults With Down Syndrome.” Frontiers in Psychology 9: 2730. 10.3389/fpsyg.2018.02730. 30705655 PMC6344413

[jar13326-bib-0051] Stedman, A. , B. Taylor , M. Erard , C. Peura , and M. Siegel . 2019. “Are Children Severely Affected by Autism Spectrum Disorder Underrepresented in Treatment Studies? An Analysis of the Literature.” Journal of Autism and Developmental Disorders 49: 1378–1390. 30536112 10.1007/s10803-018-3844-y PMC6450830

[jar13326-bib-0052] Strømme, P. , P. G. Bjømstad , and K. Ramstad . 2002. “Prevalence Estimation of Williams Syndrome.” Journal of Child Neurology 17, no. 4: 269–271. 10.1177/088307380201700406. 12088082

[jar13326-bib-0053] Tager‐Flusberg, H. , and C. Kasari . 2013. “Minimally Verbal School‐Aged Children With Autism Spectrum Disorder: The Neglected End of the Spectrum.” Autism Research 6, no. 6: 468–478. 24124067 10.1002/aur.1329 PMC3869868

[jar13326-bib-0054] Van Herwegen, J. , E. Farran , and D. Annaz . 2011. “Item and Error Analysis on Raven's Coloured Progressive Matrices in Williams Syndrome.” Research in Developmental Disabilities 32, no. 1: 93–99. 10.1016/j.ridd.2010.09.005. 20971610

[jar13326-bib-0055] Wilson, C. , L. Sitbon , B. Ploderer , J. Opie , and M. Brereton . 2020. “Self‐Expression by Design: Co‐Designing the ExpressiBall With Minimally‐Verbal Children on the Autism Spectrum.” In *Proceedings of the 2020 CHI Conference on Human Factors in Computing Systems*, 1–13. 10.1145/3313831.3376171.

