# Supplementary material for: Perceptual Experiences of Autistic People With an Intellectual Disability and People With Williams Syndrome: A Reflexive Thematic Analysis
Source: J Appl Res Intellect Disabil. 2024 Nov 29;38(1):e13326. doi: 10.1111/jar.13326 (PMC11605499; doi:10.1111/jar.13326)
Supplement: Supplementary file 1 — Data S1. Supporting information. [file JAR-38-e13326-s001.docx]

**Supplementary Materials – Interview Rubric**

**Interview schedule**

**5 min - Greeting and familiarisation**

- Warm welcome.
  - *Thank you so much for meeting with us and having time to talk to us. Welcome [Trusted Adult] and thanks for being there for [participant].*
  - Check in on how participant is doing today, is today still ok to chat?
- Introduce self
  - *I am [name]. I work in a university called [name]. I am a scientist who asks people questions about being distracted, and being focused. I like thinking about how they feel, see, hear, smell and taste.. and think.*
  - *I wrote I like I like [....] in my Research Passport [show own passport]*

**5 minutes- Consent and Research Passport**

- *Can you remember seeing our video?*
  - What helped you to choose to take part?
  - *Did you want to know anything else after you watched it?*
  - Re-go over options to stop/ take a break and that we don’t mind if they choose not to talk to us anymore.
- Show own and participant’s Research Passport
  - *Thank you for telling us about yourself in the research passport it was great to find out more about you.*

**About focus and distraction (10 minutes)**

- Introduce focus and distraction

*As part of the research we do, we want to find out how different people focus or are distracted. Some people find it easy to do things like watch a film and other people get distracted maybe because someone is being noisy or walks through the room. Everyone is different.*

*To find out more about how people focus and take in the world around them we would like to ask you some questions.*

- - When you go to [work placement] do you have specific jobs you are in charge of?/ *(Focus/ ability on mundane or jobs that aren’t a specific interest)*
  - What jobs do you like doing? What jobs do you not like doing?
  - Do you find it easier to get jobs done working with other people or working on your own?
  - Can you talk to someone and get a job done? (For instance can you do some [tasks] at the same time as chatting? Or do you start chatting and then not [work on task]?)
  - At [work placement] are there set times you have a tea break or lunch break? Do you think there should be more breaks at [work placement]?
  - How do you plan which tasks you will do at [work placement]?
  - What is your least favourite task at [work placement]? why? Do you still do it?

**Focus and distraction with enjoyed activities:**

- we loved hearing that you enjoy [topic of interest]
- What do you think about when [doing hobby]?
- What is important about [feature of hobby]?
- What happens if you get interrupted [doing hobby]?
- Is it easy for people to get your attention when [doing hobby]?
- You like [hobby] do you ever go to [large event for hobby]?
- When you’re doing [hobby] do you use headphones or speakers?
- Do your family ever say it is too loud?
- How do you focus when other people (at home/ work) are making noise/ not doing their jobs? How does that make you feel? Why is [x noise] ok but not [x noisy place]?
- Are there any things that help you cope with [x noisy place]?
- What is easier to focus on doing [hobby] or [work placement]?
- Thank you for chatting to us!

**5 minute - Invitation back to play some games about the words you know and puzzle-games you can do.**

- We will meet again to
  - Play some games about the words you know
  - Do some puzzle-games together
- Would you like to do these games on zoom, in your home?

Ask the Participant and/or Trusted Adult to relay any later thoughts about the process and this zoom call to [researchers].
